# Design, Synthesis, and Aggregation-Induced Emission Properties of a Novel D−π–A Conjugated Molecule as a Selective Hydrazine Sensor

**DOI:** 10.1021/acsomega.5c06036

**Published:** 2025-09-25

**Authors:** Omer Cansever, Guler Yagiz Erdemir

**Affiliations:** † 37511 Gazi University, Graduate School of Natural and Applied Sciences, Ankara 06560, Türkiye; ‡ 37511 Gazi University, Faculty of Science, Department of Chemistry, Ankara 06560, Türkiye

## Abstract

In this study, a new D−π–A type conjugated molecule containing *N,N*-dimethyl donor group and cyanostilbene and dicyanovinyl acceptor groups was designed and synthesized. The molecule exhibited aggregation-induced emission (AIE) property in chloroform-hexane medium; the structure, which gave low fluorescence in solution, showed intense fluorescence under low polarity conditions. In addition, it was observed that the molecule responded selectively and sensitively only to hydrazine compared to various amine compounds (TEA, DEA, aniline, ammonia), thanks to its dicyanovinyl group. A significant hypsochromic shift occurred in the spectrum in the presence of hydrazine, and an increase in fluorescence intensity was observed along with a color change. Furthermore, tests on thin-layer chromatography (TLC) determined that the molecule showed a rapid and visually observable interaction with hydrazine. These results reveal that the designed molecule can be used both in solid-state AIE-enabled optoelectronic applications and as a hydrazine-selective chemical sensor.

## Graphical abstract

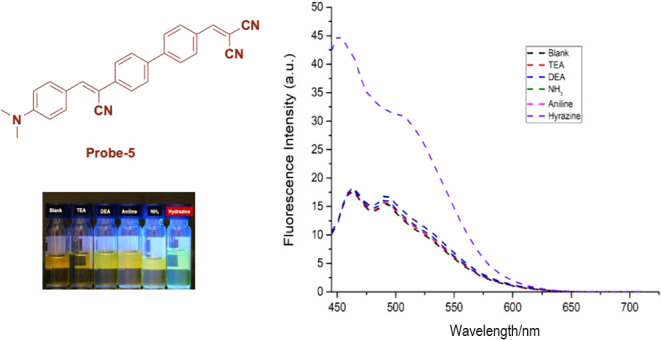

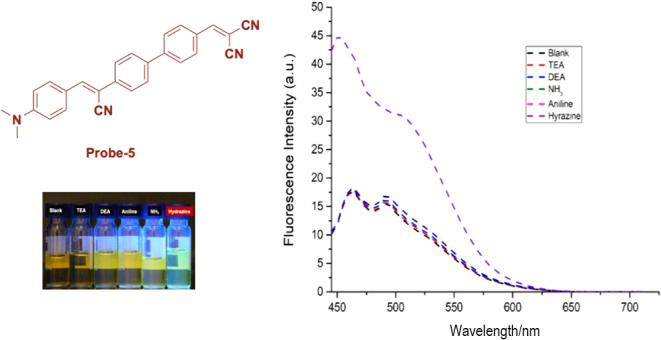

## 1 Introduction

Fluorescent molecules of the donor-π-acceptor (D-π-A) type have a broad application potential in optoelectronic materials, chemical sensors, and biological imaging due to their photophysical properties based on intramolecular charge transfer (ICT) and their sensitivity to environmental conditions.^1–5^ Such fluorescent molecules are formed by the coupling of an electron-rich donor group with an electron-poor acceptor group via a conjugated π-bridge.^5^ The D-π-A architecture facilitates ICT and allows fine-tuning of fluorescence properties.^6–11^ In this respect, it is a preferred structure, especially in the development of environmentally sensitive smart materials.^11–15^ However, in some D-π-A systems, especially in polar solvents, a weak or quenched fluorescence can be observed due to the predominance of intramolecular free rotational motions and TICT (Twisted Intramolecular Charge Transfer) states.^16^ Interestingly, such molecules can exhibit Aggregation-Induced Emission (AIE) by increasing the fluorescence intensity in the solid state or in low-solubility environments.^4,11,17,18^ In such systems, AIE arises due to restricted intramolecular motions that lead to a more geometric structure upon aggregation.^19^ In solution, AIE-enabled molecules show little fluorescence, but when exposed to weak solvents or low polarity, they can cluster and show more fluorescence.^11,20^ Generally, the designs of AIE-based molecules exhibit solid-form fluorescence based on their nonplanar aromatic skeletons that prevent π aggregation.^21^ The emission intensities of most designed AIE-based molecules generally increase at the same wavelength, and AIE studies are limited in which the molecules show a hypso- or bathochromic effect.^22^ Since these hypso- or bathochromic shifts are important in the design of optoelectronic devices, adapting such designs to AIE-based molecules and developing a suitable system will make a significant difference in this field.^23,24^ Cyanostilbene structures have a significant impact on the stability of the AIE and TICT processes due to their strong electron-attracting properties.^13,25^ Cyanostilbene structures are seen to exhibit probe properties due to their high stability, optoelectronic properties, playing an active role in AIE-based molecular designs, and being sensitive to anionic, cationic or one species.^11,26^ Hydrazine is a highly toxic and potentially carcinogenic compound that necessitates rigorous monitoring in both biological and environmental systems.^12,27–30^ Given the crucial role of detecting this small organic molecule, there is a growing need for the design of novel and highly efficient molecular probes. According to Hou and colleagues stated that they created two distinct compounds with cyanostilbene and dicyano groups, and that the dicyanovinyl group in one of these molecules is sensitive to hydrazine.^26^ Additionally, Jung et al. attached dicyano groups to tetraphenylethylene structures to create a molecule that had AIE effects and was employed as a very sensitive hydrazine probe.^11^ Taking all these factors into consideration, herein, we synthesized a conjugated D-π-A-π-A type molecule containing electron-donating *N, N*-dimethyl groups and strong electron-withdrawing properties of both cyanostilbenes and dicyano groups and observed aggregation-induced emission (AIE). It was found that the dicyano group in the molecule exhibits hydrazine probe properties due to its active vinylic structure. It was also found that the designed fluorophore offered excellent sensitivity and selectivity to detect hydrazine by exhibiting distinct spectrum shifts when hydrazine was present.

## 2 Experimental Section

### 2.1 Materials and Methods

All starting compounds were obtained from brands such as Sigma in analytical purity and were used directly. For the synthesis of the precursor compounds (*Z*)-2-(4-bromophenyl)-3-(4-(dimethylamino)­phenyl)­acrylonitrile (**3**), literature^31^ were followed. Structural characterization was established by HRMS (QTOF), FTIR, and NMR (^1^H and ^13^C-APT, recorded at 300 and 75 MHz with TMS as internal standard).

### 2.2 Synthesis of (*Z*)-2-(4-Bromophenyl)-3-(4-(dimethylamino)­phenyl)­acrylonitrile (3)

2-(4-bromophenyl)­acetonitrile (1 equiv) and 4-(dimethylamino)­benzaldehyde (1 equiv) were dissolved in 50 mL of methanol. Then, 1.2 equiv of KOH was added to this mixture, and the mixture was refluxed for 30 min, then cooled, and the solids formed were filtered. The crude product was crystallized from dichloromethane (dcm):hexane.^31^ Yellow solid. 98%, mp. 188–189 °C [190–191 °C].^31^ IR (ATR, cm^–1^): 2900, 2202, 1608, 1571.

### 2.3 Synthesis of (*Z*)-3-(4-(Dimethylamino)­phenyl)-2-(4’-formyl-[1,1’-biphenyl]-4-yl)­acrylonitrile (4)

In inert atmosphere, (*Z*)-2-(4-bromophenyl)-3-(4-(dimethylamino)­phenyl)­acrylonitrile **(3)** (1.0 mmol) were dissolved in 10:5 mL of THF:H_2_O. After that, potassium carbonate (3 mmol), (4-formylphenyl)­boronic acid (1.2 mmol) were then added to this solution, respectively. The reaction mixture was stirred for 10 min, the palladium catalyst was added, and the mixture was heated under a reflux condenser for 16 h. After the reaction was complete, it was cooled and poured into a solution of ice-cold water and extracted twice with DCM. The organic phases were collected, and the solvent was removed. The obtained derivatives **4** were purified by column chromatography on silica gel using an ethyl acetate/hexane solvent system (6/4).^32^

Yellowish-green solid. 78%, mp. 223–224 °C. IR (ATR, cm^–1^):3035, 2910, 2838, 2806, 2747, 2205, 1694,1604. ^1^H NMR (300 MHz, CDCl_3_) δ 10.07 (s, 1H), 7.97 (d, *J* = 8.1 Hz, 2H), 7.89 (d, *J* = 8.8 Hz, 2H), 7.79 (d, *J* = 8.2 Hz, 2H), 7.75 (d, *J* = 8.3 Hz, 2H), 7.69 (d, *J* = 8.5 Hz, 2H), 7.48 (s, 1H), 6.74 (d, *J* = 8.8 Hz, 2H), 3.08 (s, 6H); ^13^C-APT NMR (75 MHz, CDCl_3_) δ 191.7, 151.9, 146.2, 142.8, 139.0, 135.8, 135.4, 131.5, 130.4, 127.8, 127.5, 126.0, 121.5, 119.3, 111.6, 39.9.

### 2.4 Synthesis of (*Z*)-2-((4’-(1-Cyano-2-(4-(dimethylamino)­phenyl)­vinyl)-[1,1’-biphenyl]-4-yl)­methylene)­malononitrile (Probe-5)

To synthesize (*Z*)-2-((4’-(1-cyano-2-(4-(dimethylamino)­phenyl)­vinyl)-[1,1’-biphenyl]-4-yl)­methylene)­malononitrile (**probe-5**), compound 4 (1 mmol) and malonitrile (1.1 mmol) were dissolved in EtOH:THF (5:3) and the mixture was refluxed for 24 h. The reaction was cooled and the solids were filtered off. It was crystallized from a chloroform:hexane mixture.

Red solid. 66%, mp. 173–14 °C. IR (ATR, cm^–1^): 3030, 2916, 2797, 2223, 2205, 1607, 1589. ^1^H NMR (300 MHz, CDCl_3_) δ 7.94 (d, *J* = 8.4 Hz, 1H), 7.83 (d, *J* = 9.0 Hz, 1H), 7.75 – 7.70 (m, 2H), 7.68 (s, 1H), 7.63 (d, *J* = 8.7 Hz, 1H), 7.42 (s, 1H), 6.67 (d, *J* = 9.0 Hz, 1H). 3.02 (s, 6H); ^13^C-APT NMR (75 MHz, CDCl_3_) δ 158.3, 145.6, 142.3 137.3, 135.7, 130.8, 130.7, 129.1, 127.2, 126.9, 125.3, 125.2, 120.6, 118.5, 113.2, 112.1, 110.9, 102.6, 39.3. HRMS (ESI^+^) *m*/*z*: Calculated for C_27_H_20_N_4_ [M+H^+^]: 401.1761, found: 401.1898.

## 3 Results and Discussion

### 3.1 Molecular Structure: Design, Synthesis, and Characterization

The targeted molecule in this study was designed as a dual-functional system that exhibits both Aggregation Induced Emission (AIE) effect and offers sensitivity for hydrazine detection. The structure of the molecule was synthesized as a strong donor-π-acceptor (D-π-A) skeleton as well as a reactive dicyanovinyl (—CH=C­(CN)_2_) group that can selectively interact with nucleophilic species such as hydrazine. A stilbene unit substituted with dimethylamine was preferred as a donor moiety; it was planned to contribute to the emission behavior by effectively transferring its electron-repellent character through the conjugated system. By binding the formyl group attached to the stilbene skeleton with the electron-rich dicyanovinyl group as a result of the condensation reaction, both TICT (Twisted Intramolecular Charge Transfer) behavior was supported and a suitable binding site for analytical applications was created. Thus, the molecule was optimized to act as a chemical probe by exhibiting solvent-dependent photophysical properties and structural and optical changes, especially when interacting with hydrazine (Scheme 1).

**Scheme 1:**
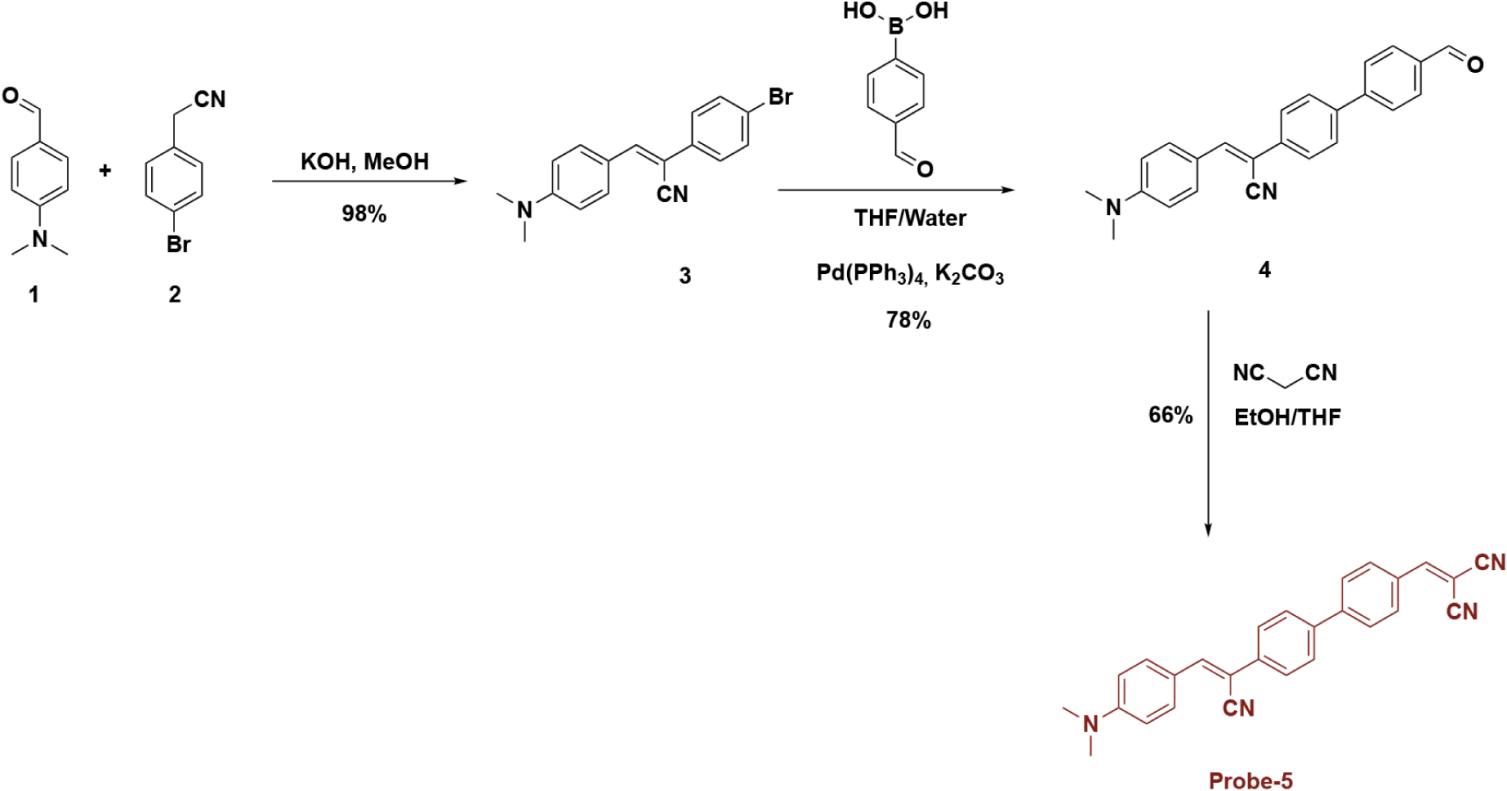
Synthesis Approach of **Probe-5**

The synthesis of the target molecule was carried out by following a three-step. First, *N,N*-dimethylbenzaldehyde and 4-bromophenylacetonitrile were subjected to a base-catalyzed Knoevenagel condensation reaction to form cyanostilbene skeleton. This intermediate product was made suitable for the Suzuki-Miyaura cross-coupling reaction due to its bromine functional group. For this purpose, the Suzuki reaction was performed with a formyl group aryl boronic acid derivative, and a formyl aryl moiety carrying the formyl group was attached to the cyanostilbene unit. In the last step, the obtained formyl-containing derivative was subjected to condensation with malononitrile under ethanol/THF solvent conditions and the molecule was attached a dicyanovinyl group. Thus, the target probe structure containing both cyanostilbene and dicyanovinyl units was synthesized. The final product was purified by chromatographic and crystallization methods and its structural confirmation was carried out by NMR, FT-IR and mass spectrometric analyses.

The structural transformations during the three-step synthesis of the molecule were followed step by step by FT-IR spectroscopy. Initially, in the cyanostilbene derivative compound obtained after the Knoevenagel condensation between *N,N*-dimethylbenzaldehyde and 4-bromophenylacetonitrile, the characteristic nitrile (−C≡N) stretching vibration was observed as a strong and sharp band around 2250 cm^–1^ in addition to aromatic ring vibrations at 1605–1571 (—C=C—). The presence of the aryl unit containing a formyl group incorporated into the structure by the Suzuki-Miyaura reaction was confirmed by the emergence of a new band around 2806, 2747, and 1690 cm^–1^ as the aldehyde (−CHO) −C-H and carbonyl stretching vibration. This band, being a signal not observed in the precursor stilbene structure, indicated that the Suzuki cross-coupling was successfully performed. In addition, in compound 4, the nitrile stretching band was observed at 2205 cm^–1^ and the stretching bands of the aromatic bands were observed around 3035 (—C= C—H) and 1600 (—C=C—) cm^–1^. In the last step, the formation of the dicyanovinyl group as a result of the condensation of the obtained aldehyde derivative with malononitrile, the emergence of an additional strong – C≡N vibration around 2220 cm^–1^ in the FT-IR spectrum, the disappearance of the carbonyl band around 1695 cm^–1^ of the vibration of the aldehyde group in this final product, and the increase in the intensity of the vibrations of the newly formed C=C—CN conjugation system in the 1580–1600 cm^–1^ band support the successful completion of the condensation reaction. This comprehensive FT-IR analysis enabled the monitoring of the chemical transformations that took place in each synthesis step and provided strong spectral evidence that the target structure was successfully synthesized (Figures S1, S2 and S5). The successful addition of 4-formylphenyl group to cyanostilbenic skeleton by Suzuki-Miyaura reaction was clearly observed in ^1^H NMR spectrum. The characteristic formyl proton belonging to the aromatic aldehyde group was observed to resonate in the region of δ ≈ 10.01 ppm, which showed that the aldehyde function was successfully incorporated into the structure by the Suzuki reaction. It was also confirmed by the appearance of the vinylic proton in the cyanostilbenic structure as a singlet around δ ≈ 7.53 ppm. The product in which aldehyde group reacted with malononitrile and formed a new dicyanovinyl bridge was clearly characterized in ^1^H NMR spectrum as a result of Knoevenagel condensation carried out with malononitrile. Complete disappearance of the signal belonging to the aldehyde proton at δ ≈ 10.01 ppm indicates that the formyl group reacted with malononitrile and condensation was completed. The appearance of a new singlet signal around δ ≈ 7.96 ppm corresponds to the new olefinic proton in the dicyanovinyl structure. The signals in the aromatic region and the NMe_2_ singlet at δ ≈ 3.02 ppm are preserved, confirming the overall skeletal integrity of the structure (Figures S3, S4, S6 and S7).

### 3.2 Photophysical Properties: AIE Study and Probe Properties

The photophysical properties of the final product (*Z*)-2-((4’-(1-cyano-2-(4-(dimethylamino)­phenyl)­vinyl)-[1,1’-biphenyl]-4-yl)­methylene)­malononitrile were investigated at a concentration of 10^–5^ M in three solvents (THF, CHCl_3_ and DMSO) having different polarities. In the UV–vis absorption spectra, two fundamental bands with similar characteristics were observed in all solvents. One of these bands was observed around 330 nm and corresponds to local π–π* transitions; the other was observed around 440 nm and attributed to intramolecular charge transfer (ICT) character. When the emission spectra were compared, it was seen that the compound gave a distinct fluorescence signal only in chloroform solution, and the maximum emission band was observed around 620 nm. In contrast, no significant emission was detected in THF and DMSO solvents (Figure 1). This result shows that the interaction of the molecule with different solvents in the excited state directly affects the fluorescence efficiency. In the UV–vis absorption spectrum of the target molecule, similar bands on 330 nm (π–π*) and 440 nm (ICT) in all solvents reveal a basically stable ground state structure, while the emission behavior changed dramatically depending on the solvents. Medium and high polarity media such as THF and DMSO promote the intramolecular charge transfer (ICT) process and promote the transition to the TICT (twisted intramolecular charge-transfer) state; this conformational phase transition strengthens the nonradiative relaxation pathways and quenches the emission to a great extent.^33,34^ It is well-known that intramolecular charge transfer (ICT) processes are promoted in polar solvents such as THF and DMSO. However, in many donor−π–acceptor (D−π–A) systems, the solvent polarity has a more pronounced effect on the emission spectra than on the absorption spectra. This is because the absorption transition typically occurs from a relatively rigid and well-defined ground state, which is less sensitive to solvent polarity. In contrast, the excited-state geometry is more relaxed and polarizable, making the emission transition more sensitive to environmental polarity. Consequently, while a red shift in emission is often observed with increasing solvent polarity (due to stabilization of the ICT excited state), the absorption maximum may remain nearly unchanged. This phenomenon is widely documented in the literature for ICT-active molecules.^35,36^ In our case, although the absorption maxima show minimal shifts across solvents, the emission data clearly support the enhancement of ICT character in polar media. On the other hand, a strong and clear emission was obtained in chloroform solution; this can be explained by the medium polarity of chloroform, its low viscosity and the existence of weak but effective interactions between the solvent and the molecule that prevent the formation of TICT.^16^ This phenomenon has also been reported in the literature for similar D−π–A systems, and it is stated that planar ICT structures are preserved in halogenated solvents such as chloroform and relaxation by radiation is prominent.^33,37^ In this context, the emission of the molecule in chloroform environment emphasizes the importance of molecule–solvent interaction and conformational effects beyond solvent polarity.

**Figure 1:**
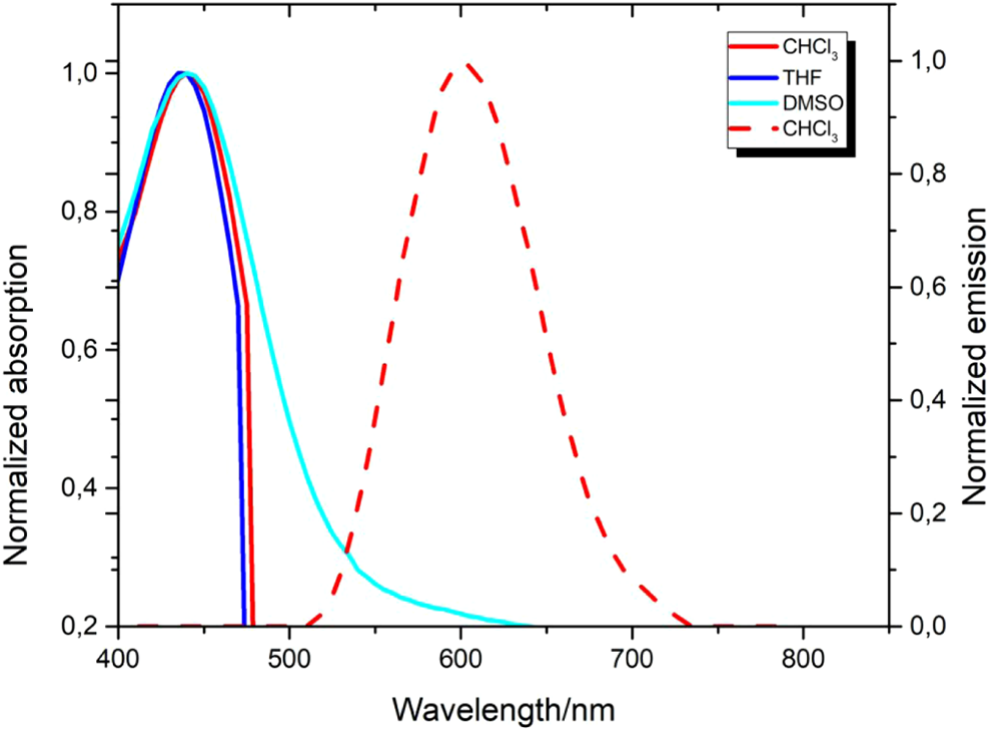
Absorption and emission spectra of **probe-5** in THF, CHCl_3_ and DMSO (excitation at 440 nm; solid-line absorption and dot-line emission).

The compound named (*Z*)-2-((4’-(1-cyano-2-(4-(dimethylamino)­phenyl)­vinyl)-[1,1’-biphenyl]-4-yl)­methylene)­malononitrile exhibited characteristic aggregation-induced emission (AIE) behavior upon increasing the hexane fraction in a chloroform solution. In good solvents such as chloroform, the compound remains molecularly dissolved and exhibits weak emission due to active intramolecular motions and a possible twisted intramolecular charge transfer (TICT) state. Upon increasing the fraction of hexane, a poor solvent, the molecule forms aggregates through enhanced intermolecular interactions. This leads to the restriction of intramolecular motions (RIM), suppression of nonradiative decay channels, and consequently a significant increase in fluorescence intensity.

Interestingly, a gradual blue shift in the emission maximum was observed, from ∼580 nm in pure chloroform to ∼540 nm in 90% hexane (Figure 2a–c). This blue shift can be attributed to the suppression of the TICT state and the enhanced contribution from locally excited (LE) states in the aggregated form. Unlike TICT states, which emit at longer wavelengths due to solvent stabilization, LE states are more rigid and often emit at shorter wavelengths, consistent with the observed hypsochromic shift.^19^ Similarly, as in some D–A type AIE systems reported in the literature, in this study, the strong donor–acceptor (D–A) structure formed by the electron donor dimethylamino group and the strong electron-withdrawing cyano and malononitrile groups promotes intramolecular charge transfer in solution. However, this charge transfer is limited in low-polarity media, and instead, more rigid and organized aggregate structures become dominant. In this context, the blue shift accompanying the increase in emission intensity reveals how the excited state character in the system changes depending on the ambient conditions and confirms the classical AIE behavior of the compound. Aggregation in AIE-active molecules typically involves the formation of nanoscale, ordered aggregates rather than random, bulk precipitation. The driving forces behind such aggregation include specific intermolecular interactions such as π–π stacking between aromatic moieties, hydrophobic interactions in low-polarity solvents, and conformational restrictions that promote the formation of rigid emissive species. These interactions facilitate the organization of molecules into defined aggregates that exhibit enhanced fluorescence, in contrast to amorphous precipitates that generally quench emission.

**Figure 2:**
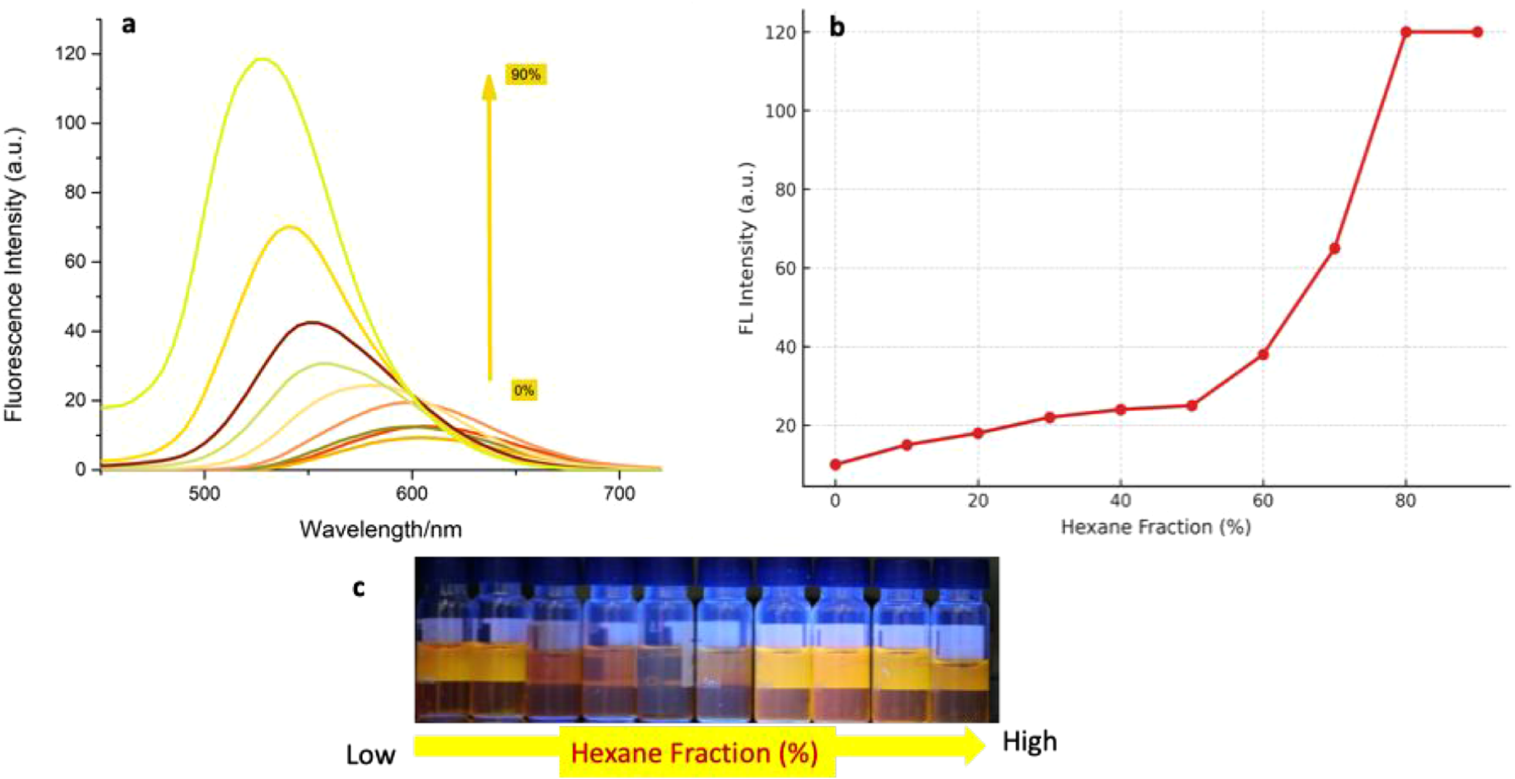
(a) Fluorescence spectra of **probe-5** (10 μM) in the CHCl_3_-hexane system with increasing hexane fractions (0–90%). (b) Plot of FI vs hexane fraction. (**c**) Photographs of fluorescence change of **probe-5** at increasing hexane contents (exposed to 365 nm lamb). λex = 440 nm.

This distinction is well-documented in the literature and supports our observation that the probe molecule, despite its low solubility in hexane, forms emissive aggregates rather than undergoing nonspecific precipitation. The increased emission intensity under low polarity conditions arises from these controlled aggregations which restrict intramolecular motions, consistent with established AIE mechanisms.^38,39^

This aggregate formation was also supported by particle size analysis performed by dynamic light scattering (DLS). DLS analysis was performed both in the solution containing only chloroform and in the solvent mixture containing 90% hexane. According to the obtained data, the average hydrodynamic diameter of the compound was determined as 244 ± 50 nm in the solution containing only chloroform. On the other hand, in the medium with low solvent polarity where the hexane ratio was increased to 90%, the hydrodynamic diameters of the compound reached 1085 ± 570 (for 68%) and 258 ± 82 (for 32%) nm (Figures S12 and S13). This significant increase clearly shows that the formation of larger aggregates occurred with the increase in intermolecular interactions. Therefore, these data support the fluorescence increase due to aggregation and confirm the AIE behavior of the compound with both photophysical and size analyses.

In the last stage of the study, the potential of the synthesized molecule (**probe**-**5**) as a selective hydrazine probe was evaluated. For the hydrazine probe, the photophysical properties of probe-5 at 3 different dielectric constants were investigated. The selectivity of the hydrazine-hydrate structure in DSMO was investigated due to the homogeneous solution environment it creates with DMSO and the environmental compatibility of the analytes in this solvent. For this purpose, (*Z*)-2-((4’-(1-cyano-2-(4-(dimethylamino)­phenyl)­vinyl)-[1,1’-biphenyl]-4-yl)­methylene)­malononitrile **(probe-5)** was dissolved in DMSO at a concentration of 10^–4^ M and then diluted with DMSO: PBS (3:1, v/v) buffer mixture to a final concentration of 10 μM. **Probe-5** was investigated for its photophysical responses to various amine and amine derivative analytes. In this context, when 50 μL of triethylamine (TEA), diethylamine (DEA), aniline, ammonia and hydrazine analytes were added to the solution separately, a characteristic spectral change was observed only in the presence of hydrazine hydrate solution (80%). No significant change was observed with other amine species, indicating that **probe-5** interacts only with hydrazine with high selectivity (Figure 3a–b). A significant hypsochromic (blue) shift of approximately 40 nm was observed in the UV–vis absorption spectrum in the presence of hydrazine. This shift suggests a change in the conjugation or ICT behavior of the molecule. The occurrence of this spectral change can probably be explained by a chemical interaction or addition reaction between hydrazine and one of the electrophilic centers of the molecule. The interaction of the **probe-5** structure with hydrazine resulted in the attack on the vinylic bridge containing the dicyano group in the molecular structure and the separation of the strong acceptor groups and their transformation into the hydrazone structure, which was confirmed by information obtained from the literature and mass spectroscopy. The mechanism of the product that will be formed as a result of this interaction is illustrated in Figure 3c. Such an interaction reduces the conjugation length of the molecule, which results in a higher energy absorption band (shorter wavelength). In addition, a significant increase in fluorescence intensity was recorded as a result of emission measurements in the presence of hydrazine. This increase can be associated with the new structure formed in the medium being more rigid, with limited conjugation and reduced nonradiative pathways. In other words, the molecule exhibits an AIE-like behavior as a result of the interaction with hydrazine.

**Figure 3:**
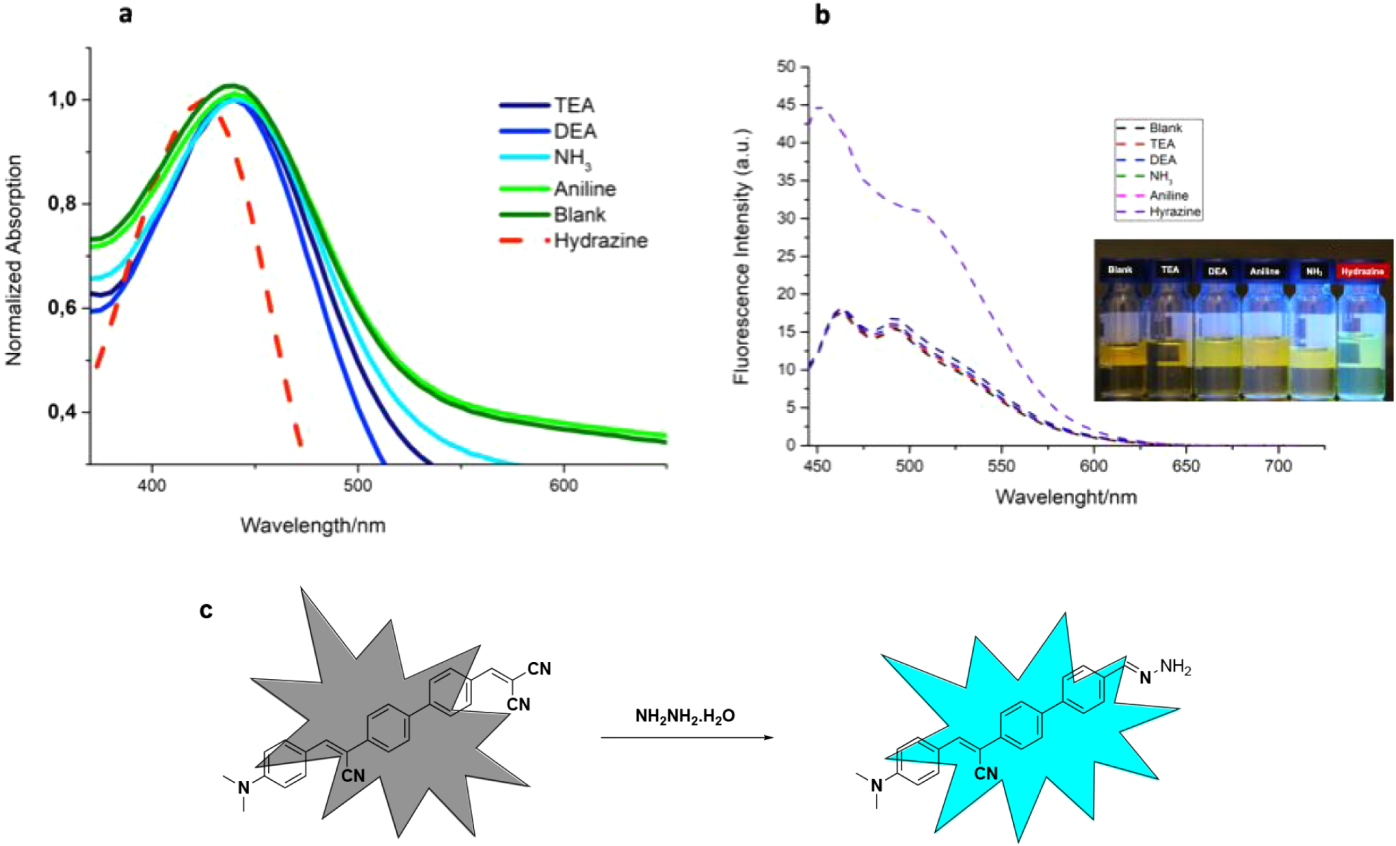
(a) Absorptions and (b) emissions of **probe-5** in different analytes are determined (c) reaction of **probe-5** as a result of interaction with hydrazine.

Within the scope of sensitivity studies, when hydrazine was added in 5 μL increments, a hypso shift of approximately 5 nm was observed in the absorption band with each addition, reaching a total shift value of 40 nm (Figure 4a–b). This dose-dependent blue shift shows that the increased interaction between the molecule and hydrazine gradually affects the conjugation structure. At the same time, this situation reveals that the sensor can be used for the detection of low amounts of hydrazine, and semiquantitative analysis can be performed. These results show that the **probe-5** can be used as a selective and sensitive hydrazine sensor, and that it can work both as a “turn-on” type sensor with especially increased fluorescence and significant absorption changes, and as an indicator that can be observed with the naked eye with color change.

**Figure 4:**
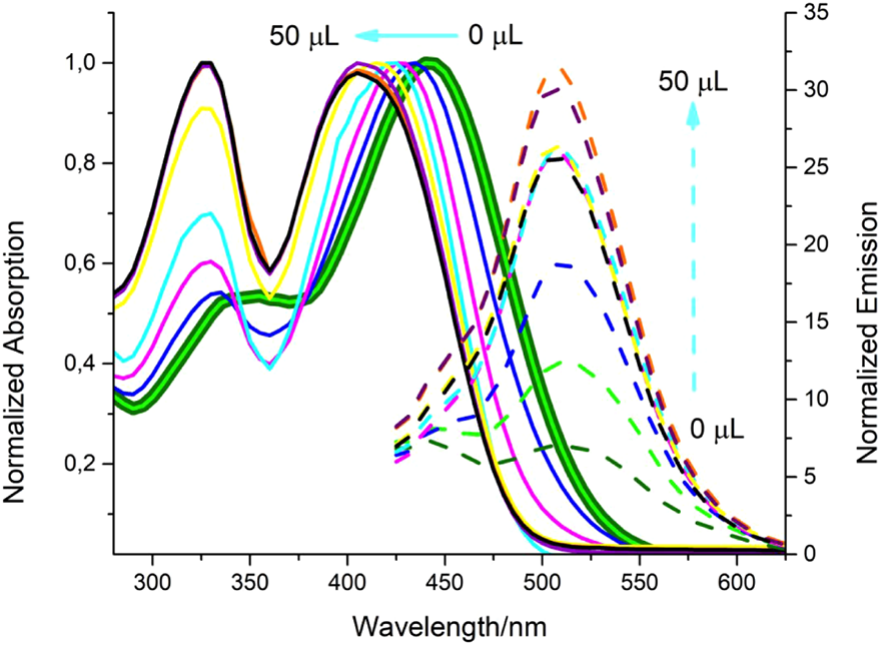
Absorption-emission changes of 10^–2^ M hydrazine hydrate solution with 5 μL additions up to 50 μL of **probe-5**.

### 3.3 Effect of pH on the Fluorescent Response of the Probe

In order to evaluate the pH effect on the detection performance of the probe, fluorescence measurements were performed in the pH range 4–11 both in the presence and absence of hydrazine. As shown in Figure 5, the probe alone did not show a significant fluorescence signal over the entire pH range. This reveals the structural stability and low background signal of the probe. However, when hydrazine was added, the fluorescence intensity increased markedly with increasing pH, especially from pH 6 and plateaued around pH 10–11.

**Figure 5:**
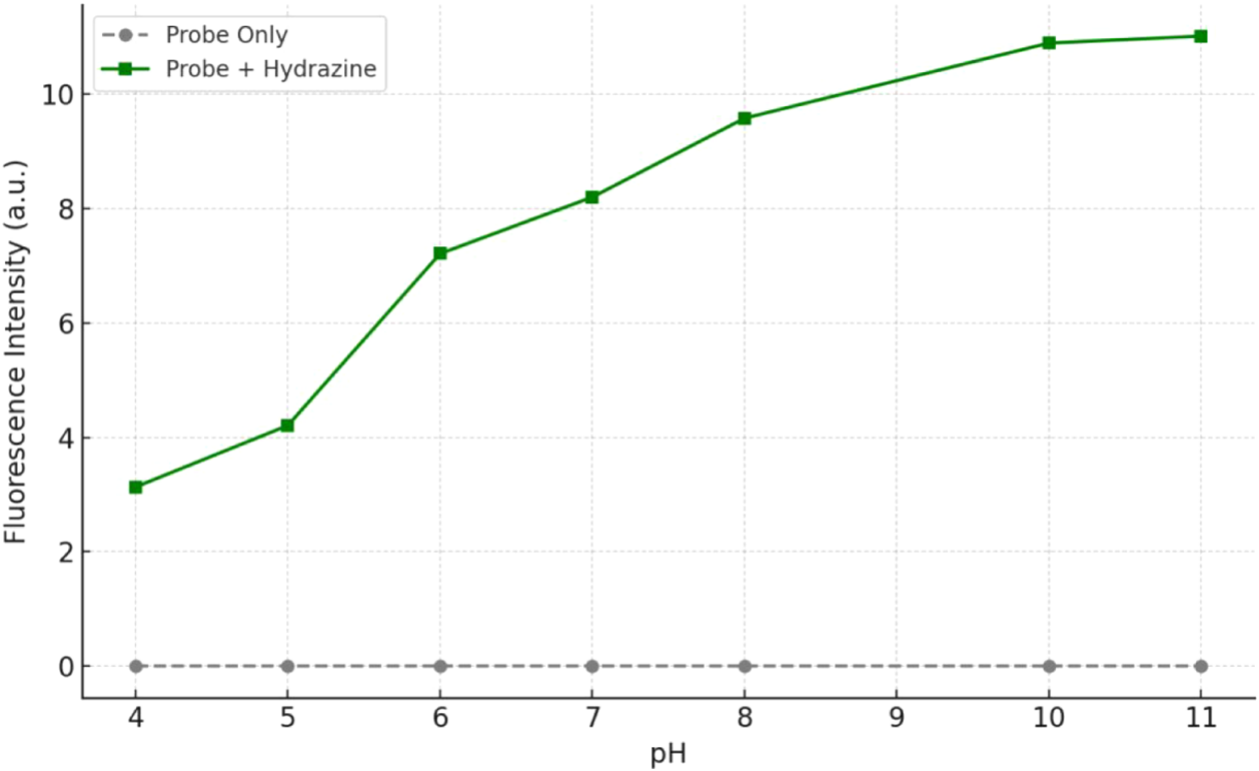
Effect of pH on the fluorescent response of **probe-5**.

This behavior is due to the fact that hydrazine is a weak base (p*K*_a_ ≈ 8.1); in mildly basic conditions it triggers fluorescence enhancement through more efficient nucleophilic attack or interaction with the probe. The low fluorescence intensity observed in acidic media (pH 4–5) is probably due to the protonation of hydrazine molecules and loss of their nucleophilic properties. These results show that the probe gives an optimum fluorescence response especially in neutral and slightly basic conditions, which is advantageous for biological and environmental applications.

For the rapid and on-site detection of the presence of hydrazine, a silica gel thin layer chromatography (TLC) plate coated with **probe-5**, which is easy to carry, was simply prepared. TLC plates were dipped in a 1 mM CHCl_3_ solution of **probe-5** and dried. These plates coated with **probe-5** were activated with the assays prepared as 10^–2^ M. While the molecules were not affected in the assays other than hydrazine, in the presence of hydrazine, the layers were affected by the silicas, and a color change was observed. In order to observe the fluorescence change, the plates were excited with a portable UV lamp at a wavelength of 365 nm (Figure 6) As a result, it was shown that a test strip sensor could be easily prepared by coating **probe-5** on a solid surface such as a silica gel TLC plate and thus the presence of hydrazine could be detected quickly and practically.

**Figure 6:**
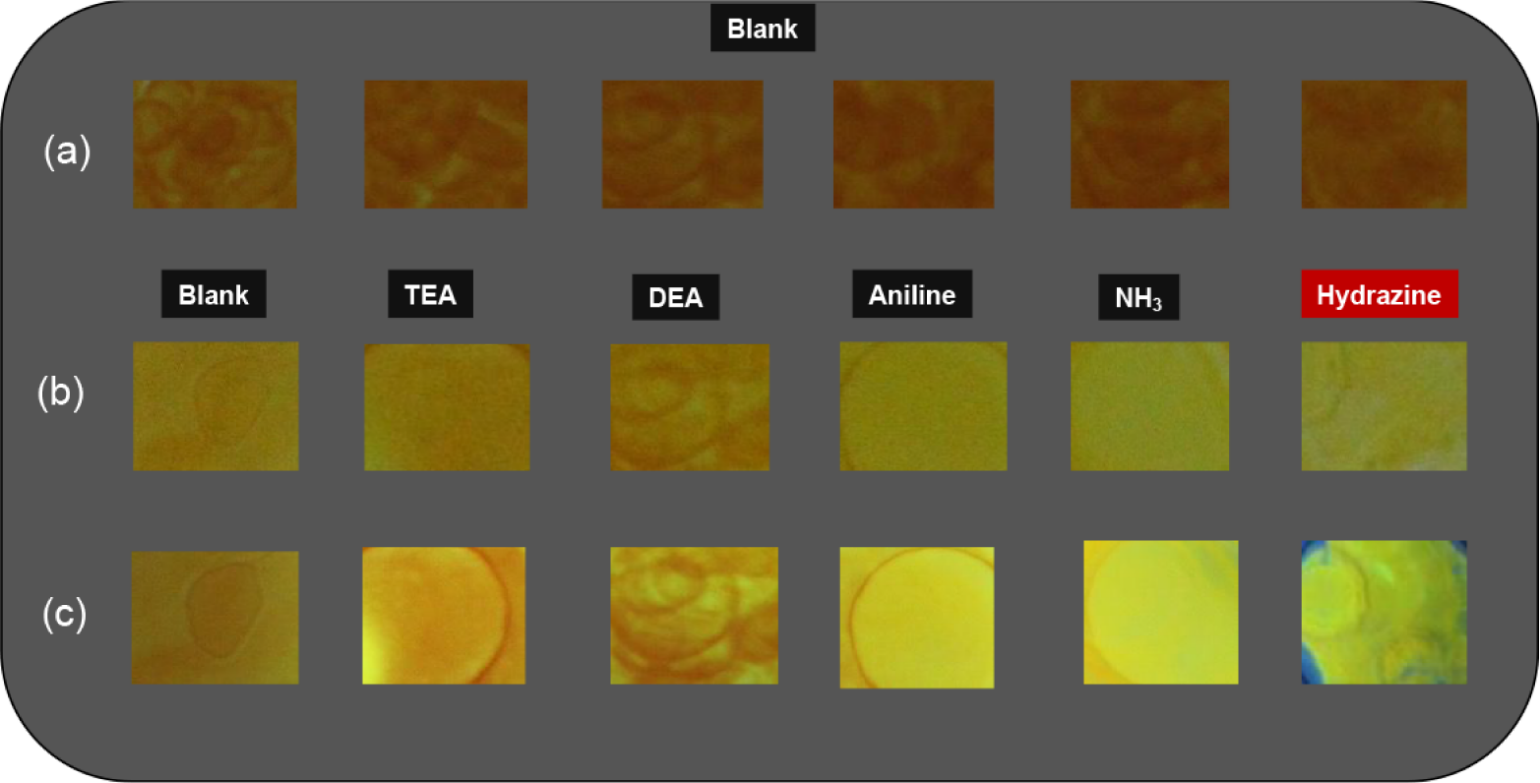
(a) Naked-eye changes of **probe-5**, (b) naked-eye color changes of **probe-5**-coated TLC plates after exposure to various amine and hydrazine, and (c) fluorescence color changes of **probe-5**-coated TLC plates after exposure to various amine and hydrazine (visualized using a portable UV lamp; excitation at 365 nm).

## 4 Conclusions

In this study, a conjugated D−π–A type fluorescent molecule containing strong electron-donating and electron-withdrawing groups was successfully designed and synthesized. The molecule exhibited suppressed fluorescence in solution, while intense emission in the aggregation state, exhibiting Aggregation-Induced Emission (AIE) property. This behavior can be explained by the restriction of intramolecular free rotational motions during aggregation and the suppression of TICT states. In addition, the acceptor unit of the molecule containing dicyanovinyl chemically interacted specifically with hydrazine, which was manifested by both a shift in the fluorescence spectrum and a color change observable by the naked eye. Thanks to its solid-state fluorescence properties, silica gel TLC plates coated with **probe-5** were used as portable and easy-to-apply test strips. These test strips showed a distinct fluorescence and color change only when exposed to hydrazine vapor, offering high selectivity and sensitivity. The developed system serves as a rapid, visual, and equipment-free identification platform in the field. In conclusion, this study demonstrates that AIE-effective, D−π–A type fluorescent molecules can be used for the selective detection of environmentally important analytes and provides valuable contributions to both fundamental photophysical research and portable sensor technologies.

## Supplementary Material



## Notes

Experimental procedures, characterization data, spectra, UV–vis and fluorescence data (PDF)
