# Supplementary material for: Design, Synthesis, and Aggregation-Induced Emission Properties of a Novel D−π–A Conjugated Molecule as a Selective Hydrazine Sensor
Source: ACS Omega. 2025 Sep 25;10(39):45671–9. doi: 10.1021/acsomega.5c06036 (PMC12508943; doi:10.1021/acsomega.5c06036)
Supplement: Supplementary file 1 [file ao5c06036_si_001.pdf]

Design, Synthesis, and Aggregation-Induced Emission Properties of a Novel D–π–A Conjugated Molecule as a Selective Hydrazine Sensor

Omer CANSEVER[1] *and Guler YAGIZ ERDEMIR*[2*]

[1]*Graduate School of Natural and Applied Sciences, Gazi University, 06560, Ankara, Türkiye*

[2]*Department of Chemistry, Faculty of Science, Gazi University, 06560, Ankara, Türkiye*

Corresponding author: Güler YAĞIZ ERDEMİR

guleryagiz@gazi.edu.tr

CONTENTS

Material Method 2

Synthesis of (Z)-2-(4-bromophenyl)-3-(4-(dimethylamino)phenyl)acrylonitrile (3)...............2

Synthesis of (Z)-3-(4-(dimethylamino)phenyl)-2-(4'-formyl-[1,1'-biphenyl]-4-yl)acrylonitrile (4)..3

Synthesis of (Z)-2-((4'-(1-cyano-2-(4-(dimethylamino)phenyl)vinyl)-[1,1'-biphenyl]-4-yl)methylene)malononitrile (probe-5) 3

Copies of FTIR, ^{1}H NMR, ^{13}C NMR spectra of the compounds 4

Results of DLS 9

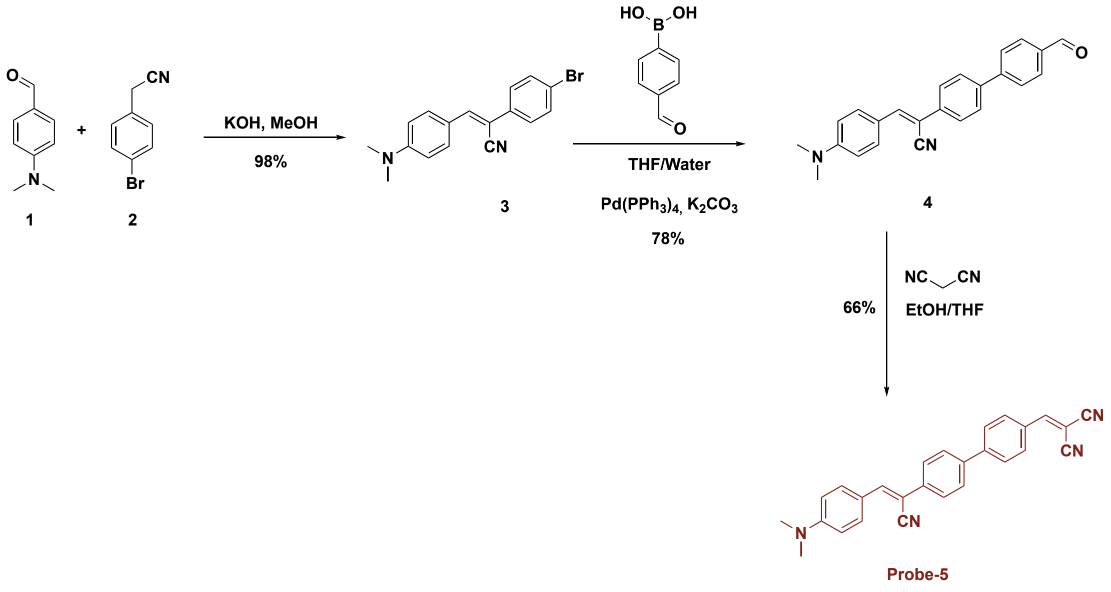

Scheme 1. Synthesis approach of **probe-5**

Material Method

All starting compounds were obtained from brands such as Sigma in analytical purity and were used directly. The reagent-grade solvents used in the purification step were purified before use. The solvents used in the reaction medium were provided in analytical purity and purified when necessary. For the synthesis of the precursor compounds (*Z*)-2-(4-bromophenyl)-3-(4-(dimethylamino)phenyl)acrylonitrile (**3**), literature[32] were followed. Thin-layer chromatography (TLC), which was carried out on aluminum plates covered with silica gel 60 F254 was used to track the development of the reaction. Column chromatography using Kieselgel 60 was used to purify the product. Mass spectrometry (using HRMS-TOF or HRMS-QTOF), infrared (IR), and nuclear magnetic spectroscopy (^{1}H and ^{13}C-APT NMR) were used to confirm the identity and purity of the produced compounds. Using TMS as the internal standard, ^{1}H and ^{13}C-APT NMR spectra were recorded on devices operating at 300 and 75 MHz, respectively. The melting points of the derivatives were recorded by the STUART (SMP-30) apparatus. Fourier-transform infrared (FTIR) spectra were screened on a Perkin-Elmer Spectrum 100 FT-IR spectrophotometer in the range of 4000–500 cm^{-1}.

Synthesis of (Z)-2-(4-bromophenyl)-3-(4-(dimethylamino)phenyl)acrylonitrile (3)

2-(4-bromophenyl)acetonitrile (1 equiv.) and 4-(dimethylamino)benzaldehyde (1 equiv.) were dissolved in 50 mL of methanol. Then, 1.2 equiv. of KOH was added to this mixture, and the mixture was refluxed

for 30 min, then cooled, and the solids formed were filtered. The crude product was crystallized from dichloromethane (dcm):hexane.

Yellow solid. 98%, mp. 188-189 °C [Lit:190-191 °C]. IR (ATR, cm^{-1}): 2900, 2202, 1608, 1571.

Synthesis of (Z)-3-(4-(dimethylamino)phenyl)-2-(4'-formyl-[1,1'-biphenyl]-4-yl)acrylonitrile (4)

In inert atmosphere, (*Z*)-2-(4-bromophenyl)-3-(4-(dimethylamino)phenyl)acrylonitrile **(3)** (1.0 mmol) were dissolved in 10:5 mL of THF:H_2O. After that, potassium carbonate (3 mmol), (4-formylphenyl)boronic acid (1.2 mmol) were then added to this solution, respectively. The reaction mixture was stirred for 10 minutes, the palladium catalyst was added, and the mixture was heated under a reflux condenser for 16 hours. After the reaction was complete, it was cooled and poured into a solution of ice-cold water and extracted twice with DCM. The organic phases were collected, and the solvent was removed. The obtained derivatives **4** were purified by column chromatography on silica gel using an ethyl acetate/hexane solvent system (6/4).

Yellowish-green solid. 78%, mp. 223-224 °C. IR (ATR, cm^{-1}):3035, 2910, 2838, 2806, 2747, 2205, 1694,1604. ^{1}H NMR (300 MHz, $CDCl_3$) δ 10.07 (s, 1H), 7.97 (d, *J* = 8.1 Hz, 2H), 7.89 (d, *J* = 8.8 Hz, 2H), 7.79 (d, *J* = 8.2 Hz, 2H), 7.75 (d, *J* = 8.3 Hz, 2H), 7.69 (d, *J* = 8.5 Hz, 2H), 7.48 (s, 1H), 6.74 (d, *J* = 8.8 Hz, 2H), 3.08 (s, 6H); ^{13}C-APT NMR (75 MHz, $CDCl_3$) δ 191.7, 151.9, 146.2, 142.8, 139.0, 135.8, 135.4, 131.5, 130.4, 127.8, 127.5, 126.0, 121.5, 119.3, 111.6, 39.9.

Synthesis of (Z)-2-((4'-(1-cyano-2-(4-(dimethylamino)phenyl)vinyl)-[1,1'-biphenyl]-4-yl)methylene)malononitrile (probe-5)

To synthesize (Z)-2-((4'-(1-cyano-2-(4-(dimethylamino)phenyl)vinyl)-[1,1'-biphenyl]-4-yl)methylene)malononitrile (**probe-5**), compound 4 (1 mmol) and malonitrile (1.1 mmol) were dissolved in EtOH:THF (5:3) and the mixture was refluxed for 24 h. The reaction was cooled and the solids were filtered off. It was crystallized from a chloroform:hexane mixture.

Red solid. 66%, mp. 173-14 °C. IR (ATR, cm^{-1}): 3030, 2916, 2797, 2223, 2205, 1607, 1589. ^{1}H NMR (300 MHz, $CDCl_3$) δ 7.94 (d, *J* = 8.4 Hz, 1H), 7.83 (d, *J* = 9.0 Hz, 1H), 7.75 – 7.70 (m, 2H), 7.68 (s, 1H), 7.63 (d, *J* = 8.7 Hz, 1H), 7.42 (s, 1H), 6.67 (d, *J* = 9.0 Hz, 1H). 3.02 (s, 6H); ^{13}C-APT NMR (75

MHz, $CDCl_3$) δ 158.3, 145.6, 142.3 137.3, 135.7, 130.8, 130.7, 129.1, 127.2, 126.9, 125.3, 125.2, 120.6, 118.5, 113.2, 112.1, 110.9, 102.6, 39.3. HRMS (ESI^+) m/z: Calculated for $C_{27}H_{20}N_4$ $[M+H^+]$: 401.1761, found: 401.1898

Copies of FTIR, ^{1}H NMR, ^{13}C NMR spectra of the compounds

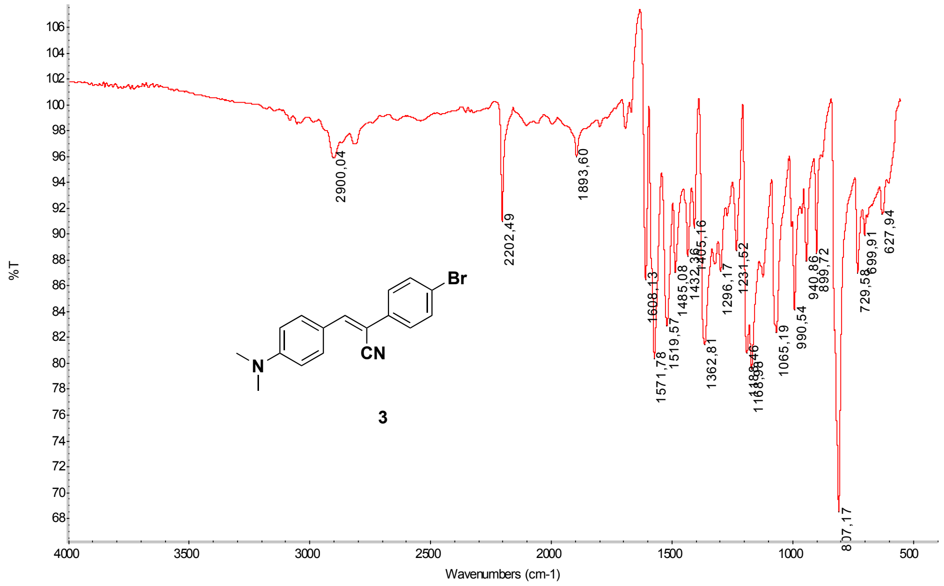

Figure S1. FTIR spectra of compound **3**

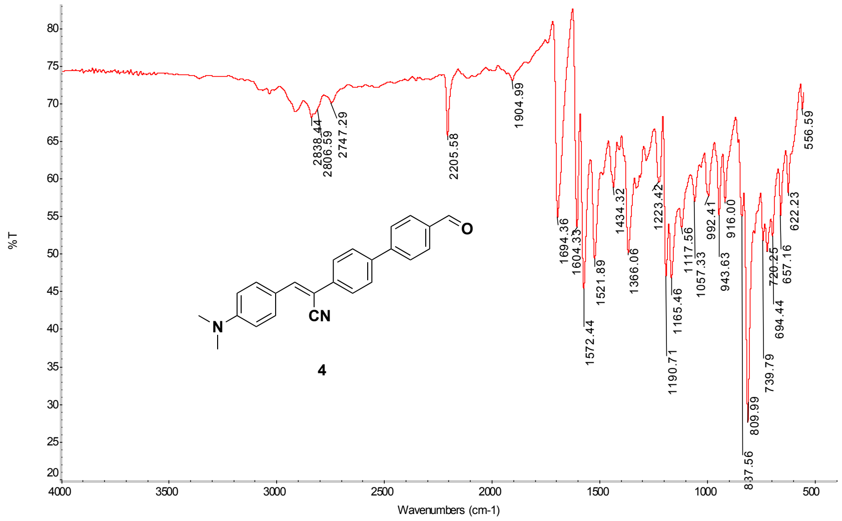

Figure S2. FTIR spectra of compound **4**

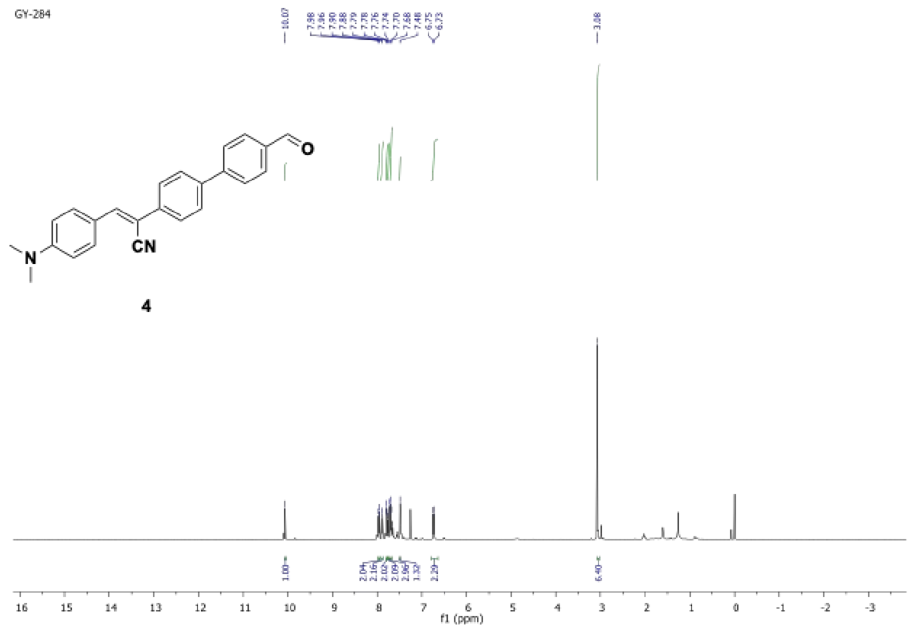

Figure S3. ^{1}H NMR spectra of compound **4**

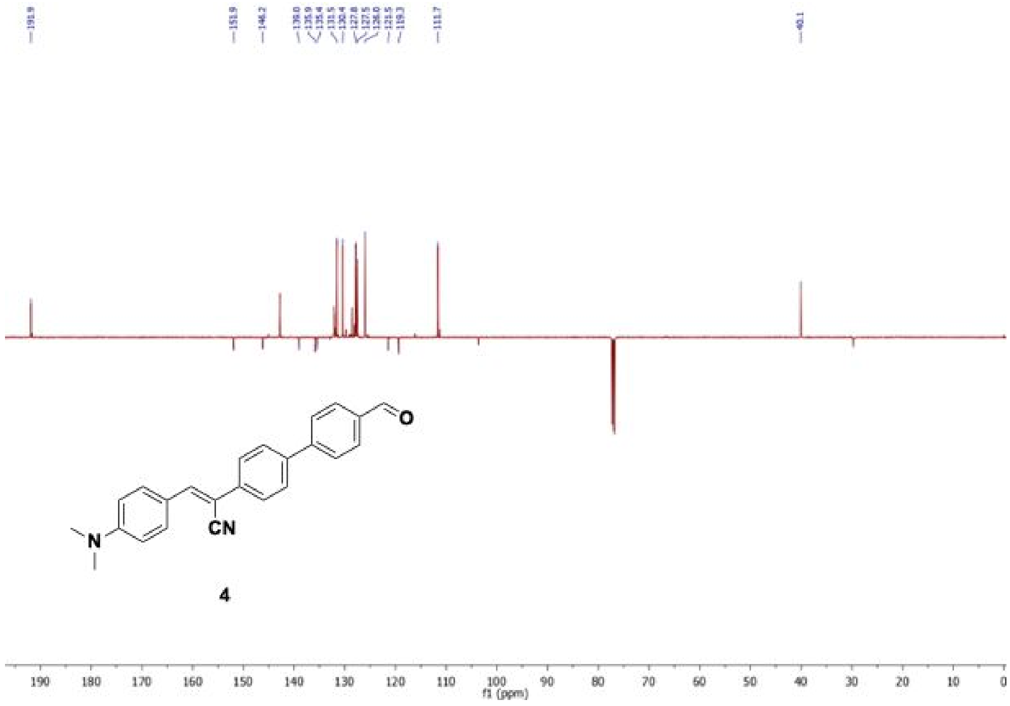

Figure S4. ^{13}C NMR spectra of compound **4**

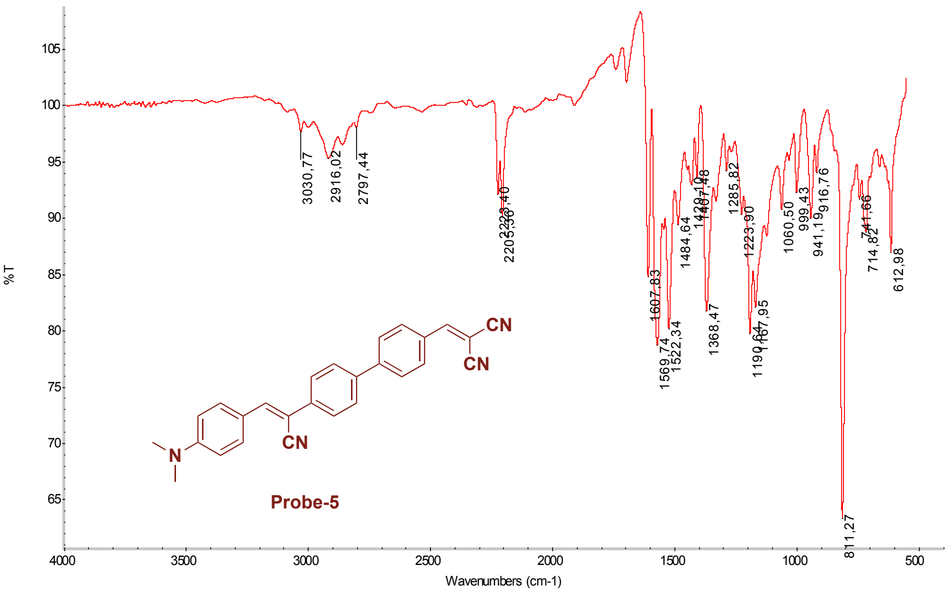

Figure S5. FTIR spectra of compound **probe-5**

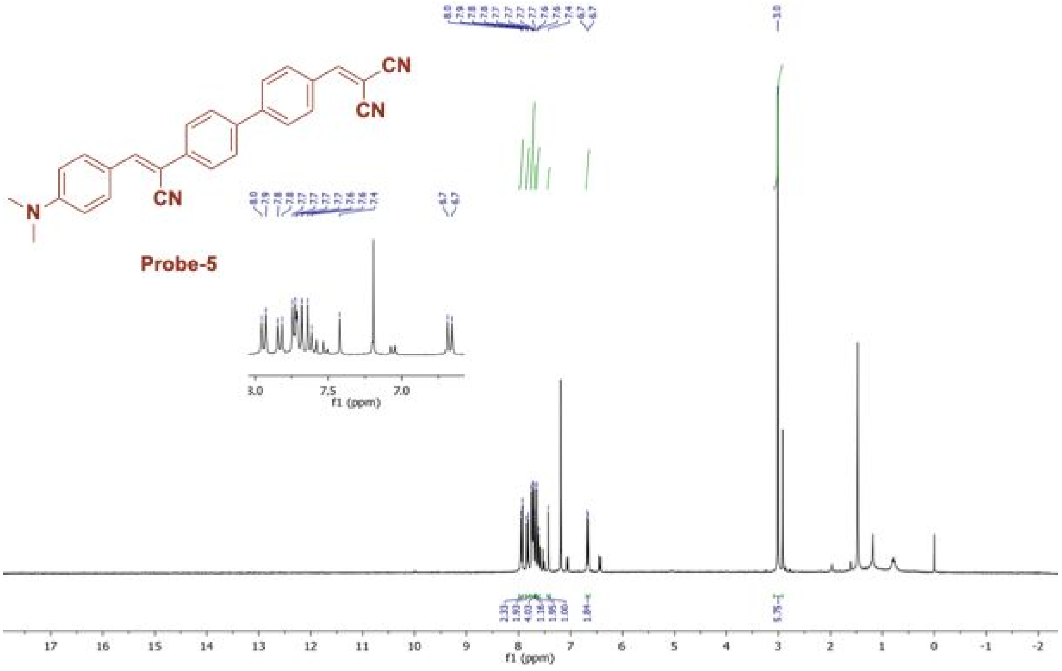

Figure S6. ^{1}H NMR spectra of compound **probe-5**

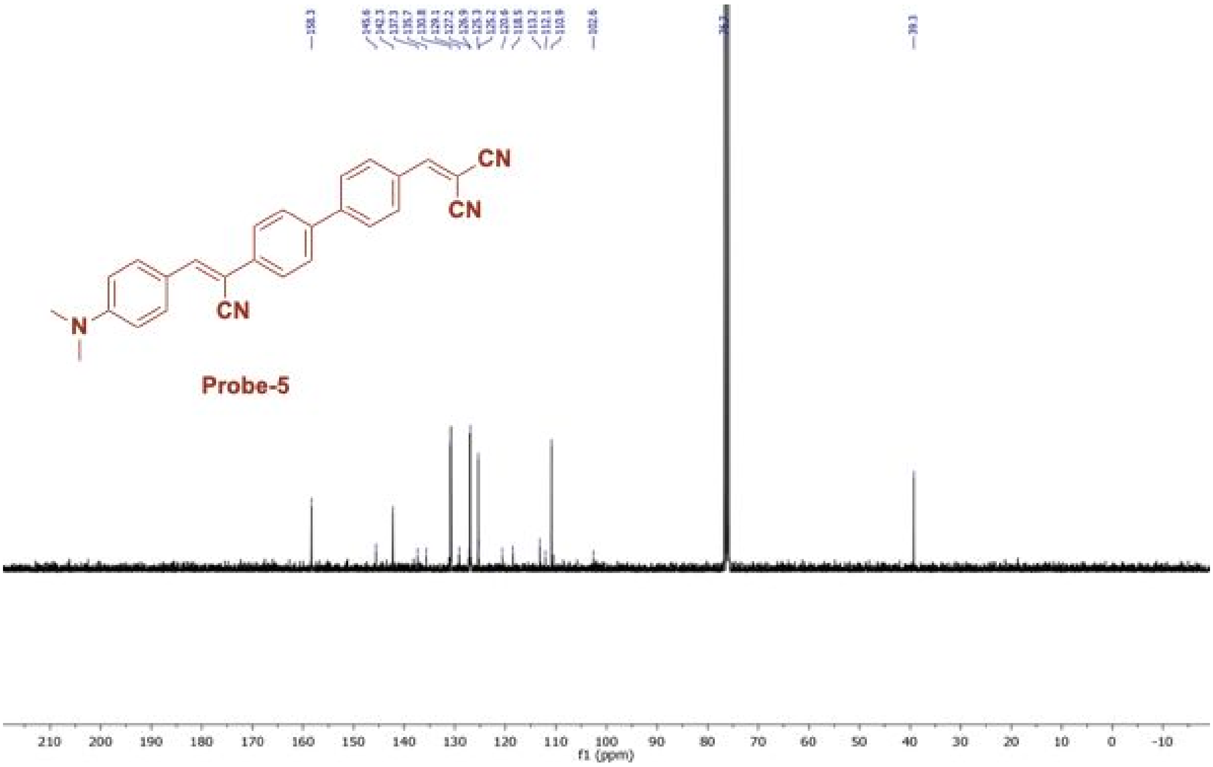

Figure S7. ^{13}C NMR spectra of compound **probe-5**

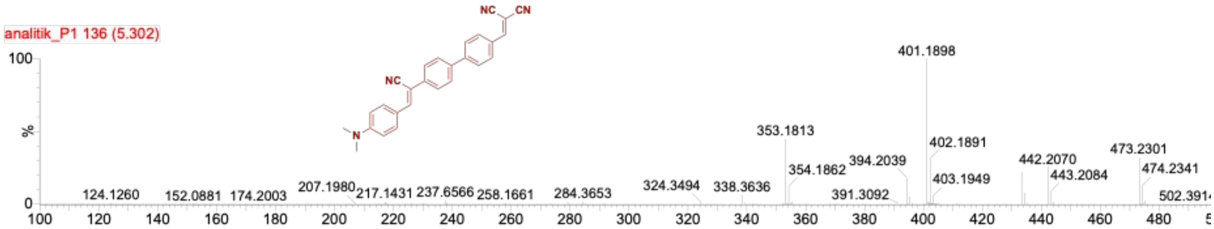

Figure S8 HRMS spectrum of **probe 5**

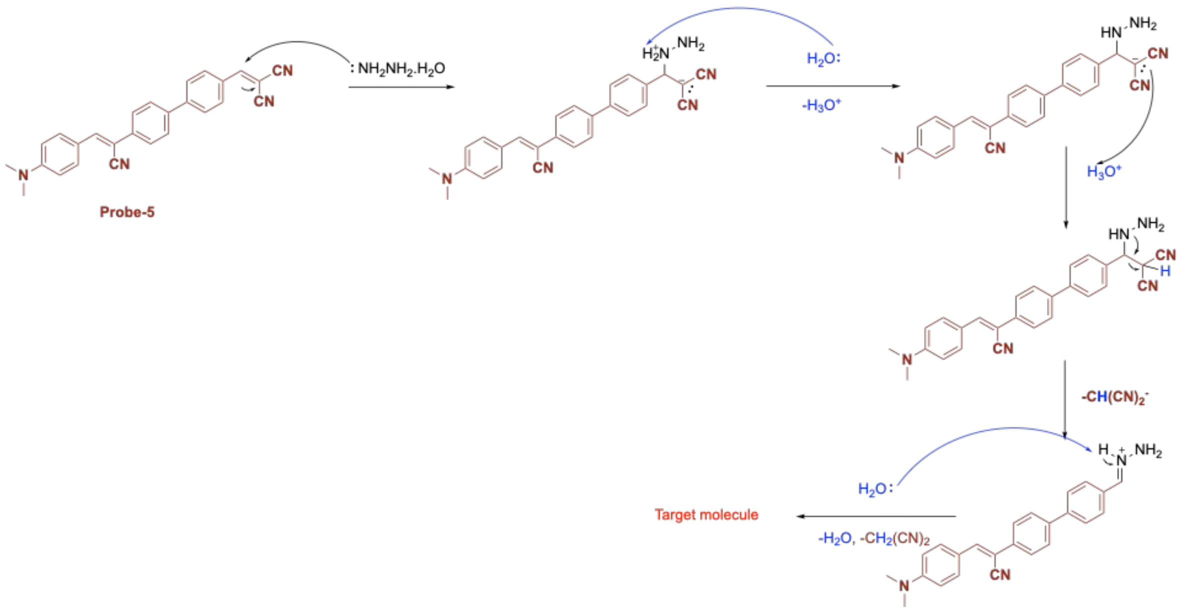

Figure S9. Possible mechanism between **probe 5** and hydrazine

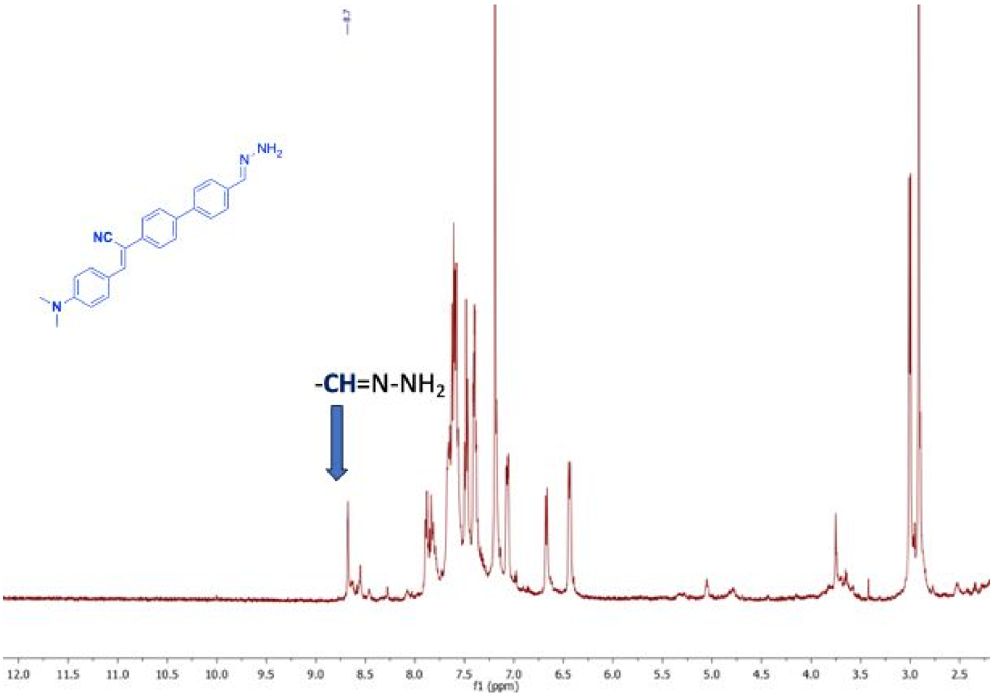

Figure S10 ^{1}H NMR spectrum of **probe 5** after interaction of hydrazine

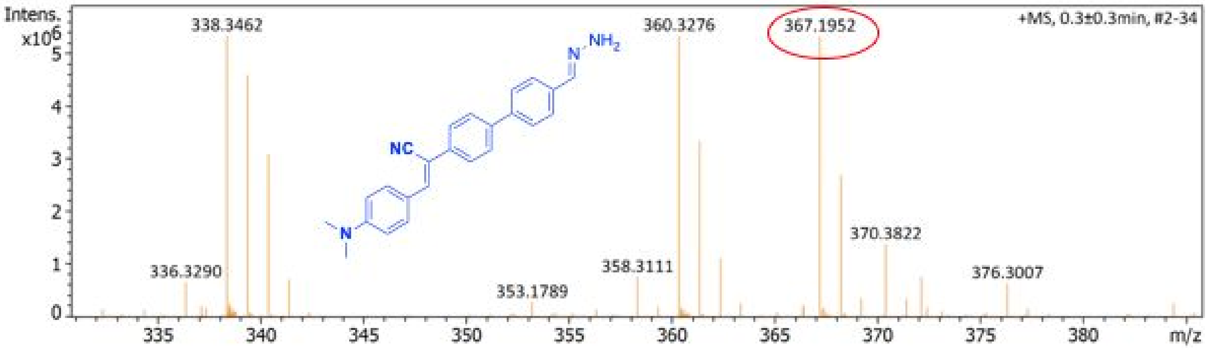

Figure S11 HRMS spectrum of **probe 5** after interaction of hydrazine

Results of DLS

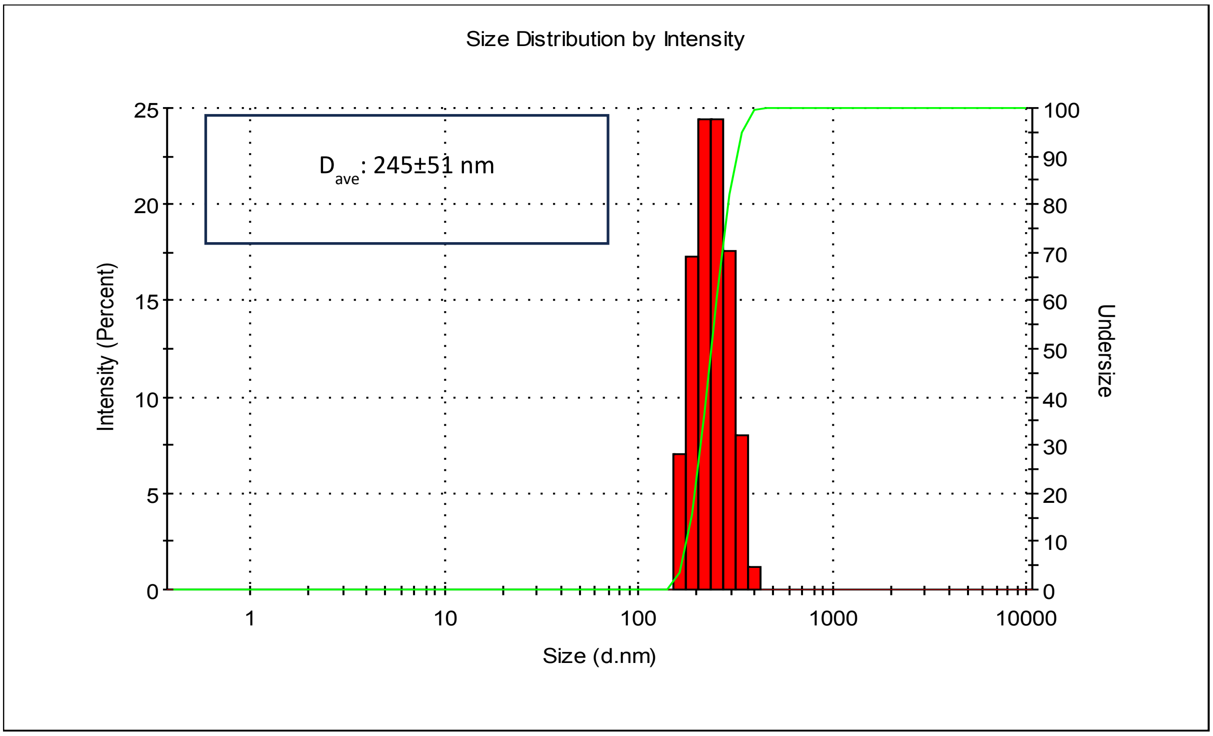

Figure S12. DLS for **probe 5** in chloroform

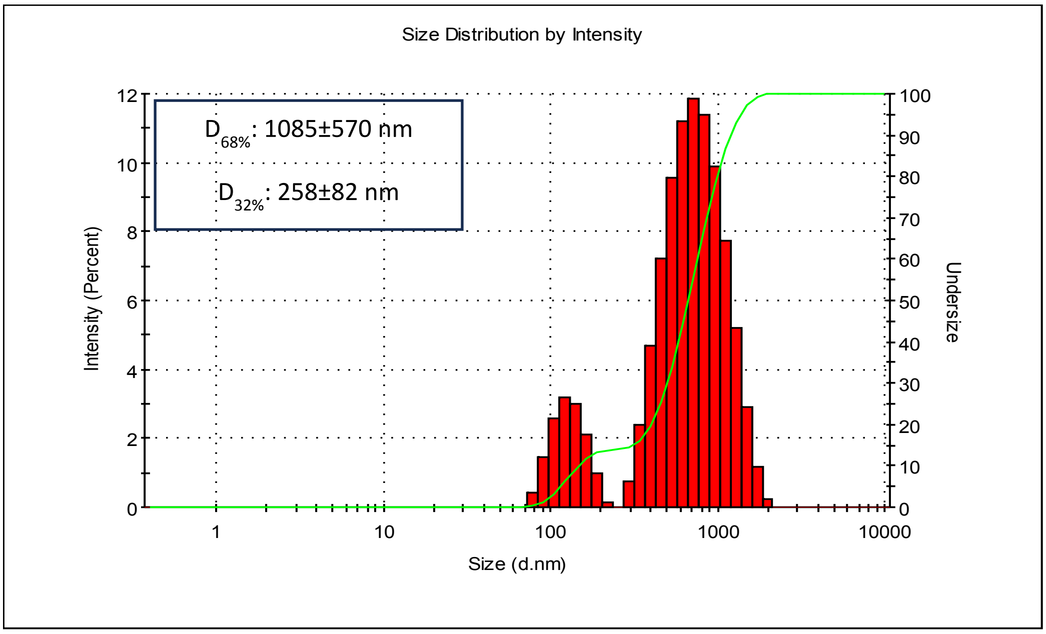

Figure S13. DLS for **probe 5** in 90%heksane:%10 chloroform
